# Supplementary material for: Asynchronous magnetic resonance elastography: Shear wave speed reconstruction using noise correlation of incoherent waves
Source: Magn Reson Med. 2022 Oct 27;89(3):990–1001. doi: 10.1002/mrm.29502 (PMC9792433; doi:10.1002/mrm.29502)
Supplement: Supplementary file 1 — DATA S1 MATLAB code used to generate the numerical simulations of Figure 1 [file MRM-89-990-s002.zip › k-Wave/helpfiles/fitPowerLawParams.html]

fitPowerLawParams :: Functions (k-Wave)


# fitPowerLawParams

Fit power law absorption parameters for highly absorbing media.

## Syntax

```
[a0_fit, y_fit] = fitPowerLawParams(a0, y, c0, f_min, f_max)
[a0_fit, y_fit] = fitPowerLawParams(a0, y, c0, f_min, f_max, plot_fit)
```

## Description

`fitPowerLawParams` calculates the absorption parameters that
should be defined in the simulation functions given the desired power law absorption behaviour defined by `a0` and `y`. This takes into account the actual absorption behaviour exhibited by the fractional Laplacian wave equation.

This fitting is required when using large absorption values or high frequencies, as the fractional Laplacian wave equation solved in `kspaceFirstOrder1D`, `kspaceFirstOrder2D`, `kspaceFirstOrder3D`, and `kspaceSecondOrder` no longer encapsulates absorption of the form a = a0\*f^y.

The returned values should be used to define the `medium.alpha_coeff` and `medium.alpha_power` within the simulation functions. The absorption behaviour over the frequency range `f_min:f_max` will then follow the power law defined by `a0` and `y`.

## Inputs

|  |  |
| --- | --- |
| `a0` | desired power law absorption prefactor [dB/(MHz^y cm)] |
| `y` | desired power law exponent |
| `c0` | medium sound speed [m/s] |
| `f_min` | minimum frequency of interest [Hz] |
| `f_max` | maximum frequency of interest [Hz] |

## Optional Inputs

|  |  |
| --- | --- |
| `plot_fit` | Boolean controlling whether the final fit is displayed (default = false) |

## Outputs

|  |  |
| --- | --- |
| `a0_fit` | power law absorption prefactor that should be used to define `medium.alpha_coeff` in the simulation functions |
| `y_fit` | power law exponent that should be used to define `medium.alpha_power` in the simulation functions |

## See Also

`kspaceFirstOrder1D`, `kspaceFirstOrder2D`, `kspaceFirstOrder3D`
